# Evaluation of the Relationship Between Zygomatic Process and Sphenoid Sinus Pneumatization in Cone-Beam Computed Tomography Images

**DOI:** 10.3390/diagnostics16060906

**Published:** 2026-03-18

**Authors:** Ezgi Katı, Gökçen Akçiçek, Hatice Yağmur Zengin

**Affiliations:** 1 Department of Dentomaxillofacial Radiology, Faculty of Dentistry, Hacettepe University, Sıhhiye, 06100 Ankara, Turkey; gokcen.akcicek@hacettepe.edu.tr; 2 Department of Dentomaxillofacial Radiology, Faculty of Dentistry, Dicle University, Sur, 21280 Diyarbakır, Turkey; 3 Department of Bioistatistics, Faculty of Medicine, Hacettepe University, Sıhhiye, 06100 Ankara, Turkey; yagmurzengin@hacettepe.edu.tr

**Keywords:** anatomic variation, cone-beam computed tomography, sphenoid sinus, temporal bone

## Abstract

**Background/Objectives**: Current evidence regarding the association between the temporal bone and paranasal sinus pneumatization remains limited. This study aims to investigate the potential morphological association between zygomatic process pneumatization and sphenoid sinus pneumatization using three-dimensional cone-beam computed tomography. **Methods**: Cone-beam computed tomography images from 573 individuals aged 16 to 87 years (170 males, 403 females) were evaluated in this study. Zygomatic process pneumatization was assessed in two forms: pneumatized glenoid fossa (a radiolucent defect on the glenoid fossa roof) and pneumatized articular eminence (a radiolucent defect within the articular eminence). The sphenoid sinus was classified into four major pneumatization types: conchal, presellar, sellar, and postsellar. The postsellar configuration was additionally divided into four subtypes—subdorsal, dorsal, occipital, and combined—according to its posteroanterior orientation. Lateral sphenoid sinus pneumatization was categorized into pterygoid, greater wing, full lateral (combining pterygoid and greater wing), lesser wing, and anterior types. **Results**: The analysis revealed a significant relationship between zygomatic process pneumatization and sphenoid sinus pneumatization (*p* < 0.001), where the former was detected in 64.0% of participants. The postsellar type represented the most frequent form of sphenoid sinus pneumatization (55.5%), whereas the conchal type was the rarest (1.2%). **Conclusions**: A significant correlation was observed between the zygomatic process and sphenoid sinus pneumatization, with individuals exhibiting the former tending to display more extensive sphenoid sinus pneumatization

## 1. Introduction

The term pneumatization describes the presence of air-filled cavities inside bones. Histologically, each cavity is lined by a monolayer of epithelial cells and demarcated from the osseous tissue by intervening subepithelial connective tissue [1,2]. The first detailed description of the locations of pneumatized regions in the temporal bone was provided by Tremble, who identified accessory air cells in ten distinct sites of the temporal bone, one of which was the articular eminence [3]. The zygomatic process may exhibit pneumatization involving the articular eminence (PAE) and glenoid fossa (PGF), which are regarded as accessory types of temporal bone pneumatization. Radiographically, pneumatization of the zygomatic process (PZP) manifests as a well-defined, asymptomatic radiolucency mimicking mastoid air cells. The lesion extends past the articular eminence while staying within the boundaries of the zygomaticotemporal suture, showing no cortical expansion or resorptive change [4]. Recognition of PZP holds clinical importance in selecting surgical approaches, assessing the potential spread of conditions such as tumors, infections, or fractures, and distinguishing it from other radiolucent entities, including aneurysmal bone cysts, hemangiomas, and central giant cell granulomas [5,6].

The sphenoid sinus (SS), located posteriorly among the paranasal sinuses, displays significant variation in morphology, volume, septal pattern, and the pneumatization of adjacent structures [7,8]. Infections of the sinus can easily involve adjacent structures, and, conversely, inflammation of neighboring tissues may extend to the sinus [9,10]. Anatomical investigations defining key landmarks of the SS and sella turcica region have greatly reduced intraoperative complications [11].

Computed tomography (CT) is widely accepted as the definitive imaging standard for radiologic evaluation of the paranasal sinuses [12]. Nevertheless, its application is restricted by disadvantages such as high cost, complex operation, and considerable radiation exposure. Conversely, cone-beam computed tomography (CBCT) is increasingly favored in dental practice because it enables rapid image capture, cost efficiency, and significantly lower radiation doses relative to traditional CT imaging [13].

While the pneumatization patterns of the articular eminence and sphenoid sinus have been extensively studied individually, their interrelationship has been addressed in only a limited number of investigations [14,15,16]. These few studies had methodological limitations and small sample sizes. Research investigating the association between the temporal bone and paranasal sinus pneumatization remains insufficient. This study was designed to explore the relationship between PZP and PSS, adjacent cranial structures, by analyzing a larger sample group, as well as the distribution patterns of PZP (PAE and PGF) and sphenoid sinus pneumatization types through CBCT imaging.

## 2. Materials and Methods

This retrospective investigation was approved by the Non-Interventional Clinical Research Ethics Committee of Hacettepe University (approval no.: GO 22/589; date: 7 June 2022). All methods complied with the ethical principles of the institutional and national human research committees and were consistent with the Declaration of Helsinki. Because of this study’s retrospective and observational nature, formal informed consent was not required.

Between January 2016 and October 2018, a total of 7495 CBCT scans obtained for various diagnostic purposes were reviewed. Among these, 573 scans (403 and 170 from female and male patients, respectively) satisfied the eligibility criteria and were included in the analysis. Since previous research indicated that sphenoid sinus pneumatization is typically completed by 16 years of age, the inclusion age threshold was set at ≥16 years [17]. Additional inclusion parameters were as follows:

• CBCT images suitable for diagnosis (free from technical, procedural, patient-related, or motion artifacts);
• Complete visualization of the sphenoid sinus, articular eminence, and glenoid fossa;
• Absence of any pathological, traumatic, developmental, or postsurgical alterations within the evaluated region.

Images were acquired using an i-CAT Next Generation CBCT unit (Imaging Sciences International, Hatfield, PA, USA), and image assessments were carried out with the i-CAT Vision software (version 1.9.3.14, Imaging Sciences International, Hatfield, PA, USA). CBCT scans were retrospectively retrieved from the institutional archive; they were originally acquired for routine diagnostic purposes, including temporomandibular joint evaluation, implant planning, and maxillofacial pathology assessment. To minimize potential selection bias, no images were selected based on the presence or absence of pneumatization patterns. Instead, all consecutively available images within the study period that fulfilled the predefined inclusion criteria were evaluated.

Because the images were obtained for different clinical indications, the field of view (FOV) was not standardized prospectively. However, only scans that had the articular eminence, glenoid fossa, and sphenoid sinus fully visualized were included, thereby ensuring anatomical completeness and comparability across cases. Acquisition parameters (120 kVp; 3–7 mA; voxel size: 0.2–0.3 mm) were within the standard operating range of the device and adjusted according to routine clinical protocols, resulting in comparable image quality suitable for morphologic evaluation.

Two oral and maxillofacial radiologists, with 8 and 21 years of professional experience, respectively, independently analyzed 60 randomly chosen CBCT scans, corresponding to 10.47% of the total dataset. To determine consistency, the same images were re-evaluated after a two-month interval. Observer reliability, considering both inter- and intra-examiner, was analyzed using the Kappa statistic, with the following thresholds: <0.20, poor; 0.21–0.40, fair; 0.41–0.60, moderate; 0.61–0.80, good; >0.80, very good [18]. Intra-observer reliability exceeded 0.81 across all variables, reflecting “very good” agreement. Inter-observer reliability demonstrated good concordance (κ = 0.79) for the left-sided PAE type and very good agreement for all remaining categories. Following confirmation of high reproducibility, final evaluations were performed by a single calibrated examiner to maintain analytical consistency and prevent potential variability that may arise from multiple observers during large-scale image interpretation. All CBCT datasets were interpreted by the same observer (EK), an oral and maxillofacial radiologist with eight years of experience.

### 2.1. Evaluation of PZP (PAE and PGF)

**Figure 1:**
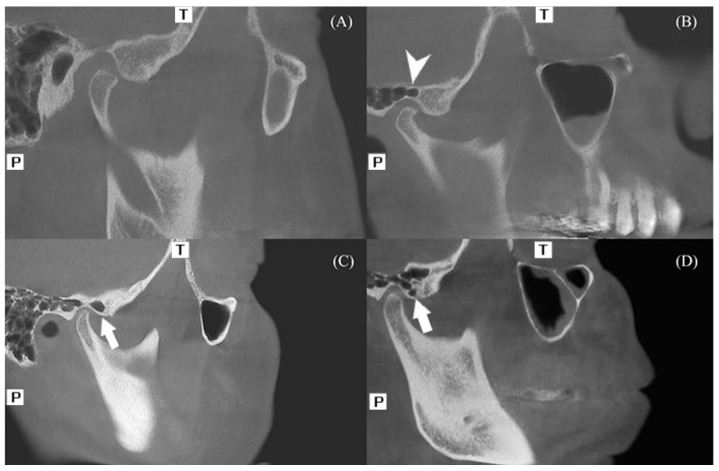
(**A**) Articular eminence and glenoid fossa without pneumatization. (**B**) Pneumatized glenoid fossa. (**C**) Unilocular-type pneumatized articular eminence. (**D**) Multilocular-type pneumatized articular eminence. (T: Top (Superior), P: Posterior).

PAE and PGF are the accessory types of temporal bone pneumatization. The characteristics described by Tyndall et al. were taken into consideration when evaluating PAE and PGF. Radiographically, these entities appear as asymptomatic radiolucent areas within the zygomatic process that mimic mastoid air cells, extending beyond the articular eminence but remaining within the limits of the zygomaticotemporal suture, without any cortical expansion or osseous destruction [4]. Each CBCT scan was examined in sagittal, coronal, and axial sections. Radiolucent cavities observed along the roof of the glenoid fossa were designated as PGF (Figure 1A,B), whereas those confined to the articular eminence were categorized as PAE. Based on their internal morphology, PAE was further divided into two subtypes:

Unilocular type: a single, well-defined, ovoid radiolucent cavity (Figure 1C);

Multilocular type: multiple, small, air cell-like cavities resembling those of the mastoid region (Figure 1D).

Despite the bilateral evaluation of PAE and PGF, the presence of PAE or PGF on at least one side was recorded as positive on a per-patient basis. Additionally, all findings—including PGF, PAE, and their respective morphological types—were documented independently for the right and left sides.

### 2.2. Evaluation of PSS

**Figure 2:**
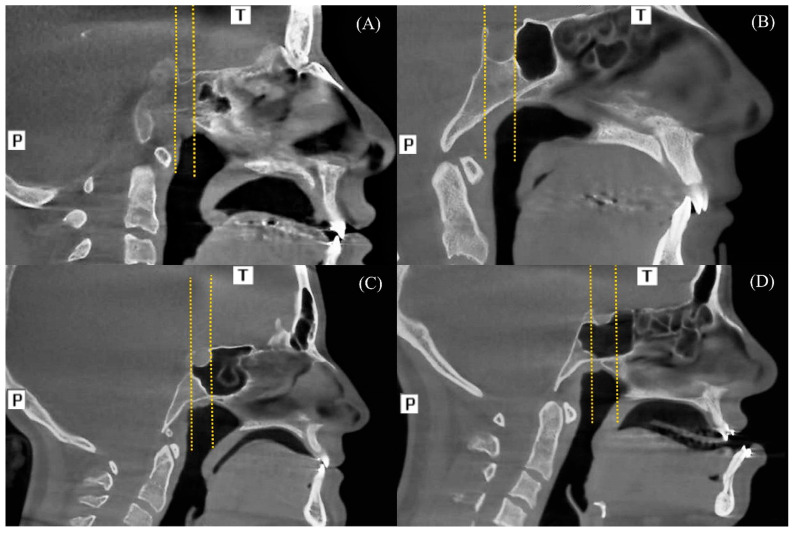
Types of pneumatized sphenoid sinus (PSS). Two vertical lines (yellow lines) were drawn along the anterior and posterior borders of the sella. (**A**) Conchal-type PSS. (**B**) Presellar-type PSS. (**C**) Sellar-type PSS. (**D**) Postsellar-type PSS. (T: Top (Superior), P: Posterior).

The classification of PSS in this study was based on a combined framework derived from the systems proposed by Hiremath et al. and Bilgir et al. [19,20]. In midsagittal reconstructions, two vertical reference lines were drawn along the anterior and posterior boundaries of the sella turcica, and the position of the posterior wall of the sphenoid sinus was evaluated relative to these lines. Four principal types of pneumatization were defined:

Conchal: The sella turcica is entirely encased in bone, with the posterior wall of the sphenoid sinus not reaching the anterior reference line (Figure 2A).

Presellar: The posterior sinus wall aligns with but does not extend beyond the anterior wall of the sella turcica (Figure 2B).

Sellar: The sphenoid sinus expands posteriorly, reaching the posterior reference line (Figure 2C).

Postsellar: The posterior sinus wall extends beyond the posterior reference plane (Figure 2D).

Postsellar pneumatization was further divided into four subtypes to reflect its inferior extension pattern. For this evaluation, two additional horizontal reference lines were drawn at the inferior margin of the sella turcica and the level of the vidian canal.

**Figure 3:**
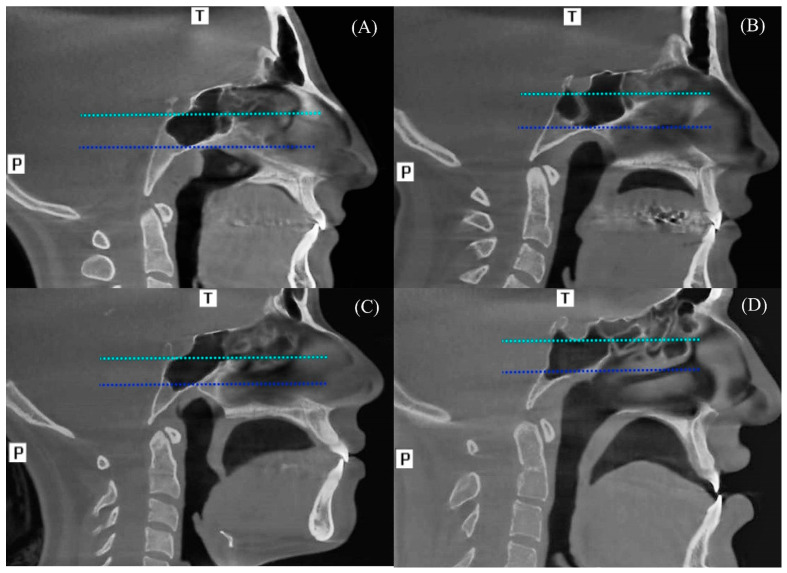
Subtypes of postsellar pneumatization. Two horizontal lines were drawn through the inferior margin of the sella turcica (turquoise line) and vidian canal (blue line) in the midsagittal section. (**A**) Subdorsal-type pneumatized sphenoid sinus (PSS). (**B**) Dorsal-type PSS. (**C**) Occipital-type PSS. (**D**) Combined-type PSS. (T: Top (Superior), P: Posterior).

Subdorsal: The sinus remains limited between the two reference lines (Figure 3A).

Dorsal: The sinus extends below the inferior margin of the sella turcica and ascends toward the dorsum sellae (Figure 3B).

Occipital: The inferior border of the sphenoid sinus reaches the level of the vidian canal but does not surpass the sella turcica’s lower border (Figure 3C).

Combined: This type represents the coexistence of both dorsal and occipital pneumatization characteristics (Figure 3D).

**Figure 4:**
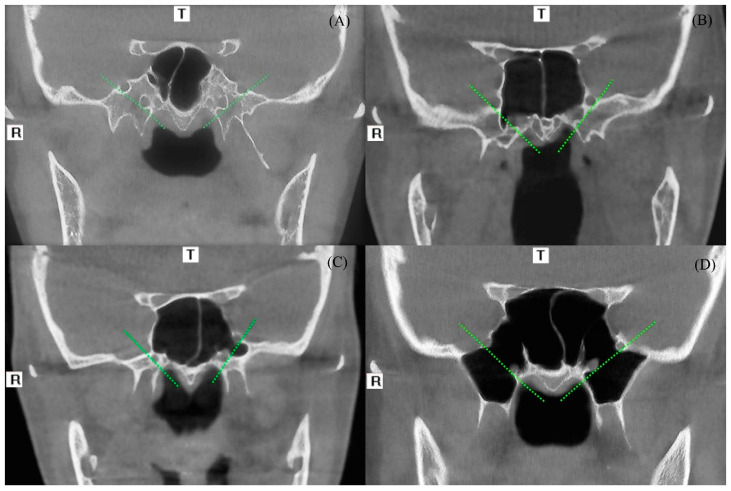
Lateral pneumatization of sphenoid sinus. In the coronal section, oblique reference lines were drawn, which connect the lateral borders of the foramen rotundum and vidian canal (green lines). (**A**) Sphenoid sinus without lateral pneumatization. (**B**) Pterygoid-type pneumatization. (**C**) Greater-wing-type pneumatization. (**D**) Full-lateral-type pneumatization. (T: Top (Superior), R: Right).

Lateral pneumatization of the sphenoid sinus was classified into three additional categories. In coronal views, oblique lines were drawn connecting the lateral borders of the foramen rotundum and the vidian canal; pneumatization extending laterally beyond these lines was assessed. If lateral extension was absent, it was recorded as “not present” (Figure 4A). All findings for the right and left SS were documented separately.

Pterygoid: Pneumatization extends through the pterygoid process and descends toward the vidian canal (Figure 4B).

Greater wing: The sinus expands laterally beneath the greater wing of the sphenoid bone (Figure 4C).

Full lateral: This type represents a combination of both pterygoid and greater wing pneumatization (Figure 4D).

**Figure 5:**
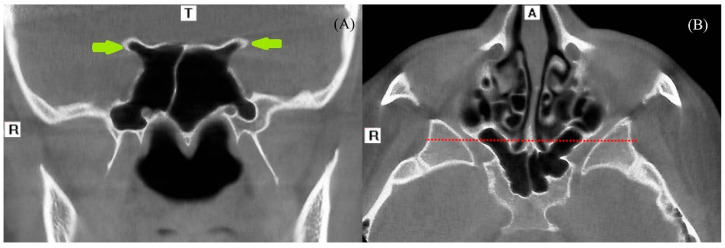
(**A**) Lesser-wing-type pneumatization (green arrows). (**B**) Anterior-type pneumatization: a horizontal reference line (red line) was drawn through the sphenoidal crest in the axial section to evaluate this type of pneumatization. (T: Top (Superior), R: Right, A: Anterior).

Additionally, the lesser wing and anterior pneumatization patterns were evaluated:

Lesser wing: In coronal images, the sinus extends anteriorly into the anterior clinoid processes (Figure 5A).

Anterior: In axial sections, a horizontal reference line was drawn through the sphenoidal crest; when the anterior boundary of the sinus crossed this line, it was classified as anterior pneumatization (Figure 5B).

### 2.3. Statistical Analysis

All statistical analyses were performed using IBM SPSS Statistics for Windows, Version 23.0 (IBM Corp., Armonk, NY, USA; 2015), and R software version 4.4.3. Categorical variables were summarized using frequency and percentage values (*n*, %). Since the sample size exceeded 50, the Kolmogorov–Smirnov test was used to examine the normality of continuous variables. Variables that did not demonstrate a normal distribution were expressed as median values with the interquartile range (P25–P75).

When the assumptions of the relevant test were fulfilled, the Pearson Chi-square test was applied to explore associations between categorical variables, while the Fisher–Freeman–Halton Exact test was used when more than 20% of the expected cell frequencies were less than 5. Cramer’s V correlation coefficient was presented as the effect size. For non-normally distributed numerical data, the Mann–Whitney U test was employed to compare differences between two independent groups. A *p*-value ≤ 0.05 was considered to indicate statistical significance. Additionally, to adjust for the effects of age and gender, binary logistic regression analysis was performed to calculate the adjusted odds ratios, where the dependent variable was PZP. When the independent variable had more than two categories with low frequencies, Firth’s logistic regression analysis was conducted to obtain robust and adjusted odds ratio estimations. Significance level was set at α = 0.05 for all analyses.

## 3. Results

PAE, PGF, and PZP were found to have a prevalence of 17.8, 63.9, and 64.0%, respectively (Table 1). The Pearson Chi-square test indicated no significant gender differences in the distribution of PZP prevalence (*p* = 0.866). The median age was 27 with PZP, and 33 without PZP. The Mann–Whitney U test results indicate that the probability of pneumatization decreases with advancing age (*p* = 0.004).

The types and characteristics of sphenoid sinus pneumatization (PSS) are presented in Table 2.

This study found that 55.5% of sphenoid sinuses exhibited postsellar pneumatization, whereas conchal-type pneumatization was identified in only 1.2% of cases.

The Fisher–Freeman–Halton exact test was employed to examine the relationship between PSS and gender, revealing no significant difference in PSS across gender (*p* > 0.05).

Chi-square analysis was employed to assess the relationship between PSS and PZP, revealing a statistically significant association, with a small effect size that approached the threshold for a moderate effect (Cramer’s V = 0.285, *p* < 0.001). A statistically significant relationship was observed between PZP and the subtypes of postsellar-type pneumatization (*p* < 0.001). The analysis of the correlation between SS lesser-wing-type pneumatization and PZP demonstrated statistical significance (*p* = 0.001). A statistically significant difference was not observed between the anterior type of PSS and PZP (*p* = 0.677) (Table 3).

To determine whether these associations were independent of potential confounders, an adjusted binary logistic regression analysis was performed, controlling for age and gender. The postsellar PSS type was used as the reference category. In the adjusted logistic regression model, postsellar sphenoid sinus pneumatization emerged as the configuration most strongly associated with PZP, while the conchal, presellar, and sellar types demonstrated significantly lower odds compared to the postsellar reference category. Additionally, lateral and lesser wing pneumatization independently increased the likelihood of PZP, whereas anterior pneumatization showed no significant association (Table 4).

These findings indicate that PZP is specifically associated with advanced posterior and lateral sphenoid sinus pneumatization patterns rather than a generalized cranial air cell phenomenon.

## 4. Discussion

The detection of zygomatic air cells has a significant impact on the diagnosis and treatment of skeletal anomalies [21]. Moreover, the potential for complications during surgical procedures is compounded by the suggestion that pneumatic air cells may facilitate the dissemination of infection to adjacent tissues [22]. It has been hypothesized that pneumatized cells in the glenoid fossa may cause intact bone tissue thinning between the cranial fossa and the temporomandibular joint (TMJ) space, which may be problematic in maxillofacial trauma [23]. In addition, it is crucial to conduct a differential diagnosis of air cells, a condition that does not require treatment. In the differential diagnosis, it is essential to evaluate radiolucent diseases, including eosinophilic granuloma, hemangioma, aneurysmal bone cyst, central giant cell granuloma, and multiple myeloma [24,25,26].

Numerous studies have investigated the prevalence of PZP, but, overall, none have revealed statistically significant differences between genders [27,28,29,30]. Similarly, this study found no statistically significant difference in the incidence of PZP between male and female populations (*p* = 0.866).

Some studies indicate that there is no association between age groups and the occurrence of PZP. Adişen et al. and Bhalchim et al. reported that the prevalence of PAE is greater in the third decade [27,31]. Similarly, in this study, the median age was 27 for participants with PZP.

Anatomical variations in pneumatization of the SS have a significant impact on surgery of the pituitary gland [32]. The surgeon can avoid complications such as hemorrhage, infection, and cerebrospinal fluid leakage by understanding the SS’s anatomical details and degree of pneumatization [33,34]. In the presence of extensive pneumatization, the risk of invasion of the internal carotid artery into the SS increases [7]. Also, due to the anatomical significance of the SS, there is a risk of severe infection-related complications [10]. For this reason, scientists have studied PSS for several years and developed various classifications.

The literature reveals a variety of PSS classification models. Therefore, although it is difficult to assess the prevalence of PSS, it is evident that conchal pneumatization is the most uncommon type [11,19,20,32,35,36,37]. In our study, conchal-type pneumatization was the rarest, found only in seven individuals (1.2%), which is consistent with the literature.

Regarding the most prevalent form of posteroanterior pneumatization, researchers have obtained conflicting results. According to some researchers, sellar-type pneumatization occurs more frequently [11,32,35,36]. Other researchers have concluded that the postsellar type is more prevalent [19,20,38]. Some of these researchers classified posteroanterior pneumatization only in three categories: conchal, presellar, and sellar, but not postsellar. We believe that this inhibits the determination of the most prevalent pneumatization type. The most prevalent forms of pneumatization in this study were sellar and postsellar, with prevalence rates of 38.4% and 55.5%, respectively, which is consistent with the literature.

This study aims to determine the prevalence of PZP, the kind and prevalence of PSS, and their interrelationship. Limited research has been published on these subjects [14,15,16]. Gibelli et al. [16] analyzed 200 CT scans to assess the various configurations of air-filled cavities in the temporal bone, specifically the glenoid fossa, petrous apex, and infralabyrinthine region, in addition to the ethmoid and sphenoid sinuses. It was found that in females, the air-filled petrous apex correlated with the air-filled anterior clinoid process, while the air-filled infralabyrinthine section correlated with the volume of the sphenoid sinus. Unlike our investigation, they studied fewer images and had a less comprehensive categorization of the sphenoid sinus. Hindi et al. [15] examined 150 CT scans from three different racial groups (Malay, Chinese, and Indian) in order to assess the level of air-filled spaces in the temporal bone and ethmoid and sphenoid sinuses. They identified a positive correlation between the pneumatization of mastoid air cells and the sphenoid sinus. In contrast to our study, they disregarded the PZP from their investigation and solely categorized the sphenoid sinus in the posteroanterior orientation. Kim et al. [14] conducted a retrospective study where they analyzed the three-dimensional reconstruction of 60 CT images. The aim was to assess the relationship between the existence of air cells in the mastoid area and the paranasal sinuses. A correlation was also identified between the dimensions of the sphenoid sinus and mastoid air cells.

While previous CT-based studies have demonstrated an association between temporal bone pneumatization and sphenoid sinus development, the specific relationship between PZP and detailed PSS patterns has not been comprehensively evaluated. Earlier investigations primarily focused on mastoid air cells or general temporal bone pneumatization and typically assessed sphenoid sinus pneumatization using limited volumetric classifications [14,15,16].

The novelty of the present study lies in several methodological and analytical aspects. First, PZP was evaluated as a distinct anatomical entity rather than as part of a group under general temporal bone pneumatization. Second, PSS was analyzed using a comprehensive classification system, including posteroanterior types (conchal, presellar, sellar, postsellar), detailed postsellar subtypes (subdorsal, dorsal, occipital, and combined), lateral extension patterns, and lesser wing pneumatization. This multidimensional approach enabled a more refined assessment of anatomical variations. Third, this study was conducted on a relatively large CBCT dataset, allowing the evaluation of high-resolution, three-dimensional images while reflecting routine dentomaxillofacial imaging practice.

Unlike previous studies that primarily demonstrated a general correlation between mastoid or temporal bone pneumatization and sphenoid sinus volume [14,15], the present findings provide evidence that PZP is specifically associated with more extensive posteroanterior pneumatization patterns (*p* < 0.001), advanced postsellar subtypes (*p* < 0.001), lateral pneumatization types (*p* < 0.001), and lesser wing involvement (*p* < 0.001). These results expand existing knowledge by identifying PZP as a potential radiological indicator of extensive sphenoid sinus pneumatization rather than merely confirming a broad temporal–sphenoid relationship.

Therefore, this study advances the current understanding by offering a more detailed morphological framework and clarifying the specific anatomical patterns through which PZP and PSS are interrelated.

The adjusted regression model confirms that the association between PZP and sphenoid sinus pneumatization is independent of age and gender and anatomically selective. In particular, lateral extension and lesser wing involvement significantly increased the likelihood of PZP, whereas anterior pneumatization showed no predictive value. This selective posterior–lateral relationship suggests a shared developmental expansion pattern rather than a generalized pneumatization process.

From a clinical standpoint, the identification of PZP on routine CBCT imaging may serve as a radiological marker of extensive postsellar and lateral sphenoid sinus expansion. Such configurations are relevant in surgical planning involving the cranial base, temporomandibular joint region, and sphenoid sinus, where increased pneumatization may be associated with cortical thinning, anatomical variation, and potential procedural risk. Therefore, the predictive value demonstrated in this study enhances preoperative anatomical assessment and contributes to a more refined understanding of cranial pneumatization morphology.

Tyndall et al. proposed that pneumatized articular eminence (PAE) may represent an extension of mastoid air cells [4]. In this study, the higher prevalence of PGF compared with PAE may be interpreted within this developmental framework. Similarly, Adışen et al. reported that individuals with PAE exhibited larger mastoid air cell volumes than those without PAE, suggesting a possible relationship between mastoid air cell pneumatization and PAE [39]. Altogether, these observations point to potential anatomical continuity between mastoid air cells and temporal bone pneumatization patterns; however, causal or developmental mechanisms cannot be established based on the current data.

The observed association between PAE and/or PGF and PSS may reflect a tendency toward more extensive cranial pneumatization patterns rather than isolated anatomical variations. However, as the present study was not designed to investigate developmental or genetic determinants, no conclusions can be drawn regarding an underlying genetic predisposition. Instead, the findings demonstrate a morphological association between different pneumatized structures of the cranial base. Further well-designed studies incorporating developmental, longitudinal, or genetic data are required to clarify the mechanisms underlying this relationship.

Although the prevalence and relationship between PSS types and PZP (PAE and PGF) were examined in detail, this study has several limitations. First, the areas of pneumatization in other temporal bone regions were excluded from this study. In the present study, only PAE and PGF, which are most familiar to dentists due to their proximity to the TMJ, were evaluated for their impact on the type of surgical intervention and the spread of infection. This precludes disclosing the connection between the general pneumatization of the temporal bone and PSS. The maxillary sinuses were excluded from this study due to the numerous anatomical variations they contain and the effect of tooth extraction on maxillary sinus pneumatization [40]. The frontal sinus was also excluded because it can only be assessed with a large field-of-view CBCT scan. Consequently, it was unfeasible to examine the correlation between the pneumatization of the zygomatic process and the extent of pneumatization in other sinuses. In addition, it was not possible to acquire a detailed anamnesis from the individuals whose images were examined because of the retrospective nature of this study. Therefore, the function of environmental factors in the development of pneumatized cells cannot be determined. More extensive research is required in this area.

## 5. Conclusions

PZP is significantly associated with advanced sphenoid sinus pneumatization patterns, particularly postsellar and lateral pneumatization. These findings suggest that PZP may serve as a radiological indicator of extensive cranial base pneumatization. Recognition of these anatomical variations may improve preoperative assessment in procedures involving the TMJ and cranial base, where extensive pneumatization may influence surgical planning and risk evaluation. Further studies are needed to clarify the developmental mechanisms underlying these associations.

## Figures and Tables

**Table 1:** Prevalence of PAE, PGF, and PZP.

	Sample (*n* = 573)
Sex (Female/Male)	403 (70.3%)/170 (29.7%)
Age *	29 (21–41)
PZP	367 (64.0%)
PAE Total	102 (17.8%)
PAE Right	61 (10.6%)
Unilocular	7 (1.22%)
Multilocular	54 (9.4%)
PAE Left	78 (13.6%)
Unilocular	13 (3.2%)
Multilocular	65 (11.3%)
PGF Total	366 (63.9%)
PGF Right	283 (49.4%)
PGF Left	325 (56.7%)

* Median (IQR: P25–P75). PAE: pneumatized articular eminence; PGF: pneumatized glenoid fossa; PZP: pneumatization of the zygomatic process.

**Table 2:** Pneumatization of sphenoid sinus types and features.

PSS Type	Conchal	7 (1.2%)
	Presellar	28 (4.9%)
	Sellar	220 (38.4%)
	Postsellar	318 (55.5%)
Subtypes of Postsellar Pneumatization	Subdorsal	165 (28.8%)
	Dorsal	47 (8.2%)
	Occipital	78 (13.6%)
	Combined	28 (4.9%)
Lateral Pneumatization	Right	259 (45.2%)
	Pterygoid	78 (13.6%)
	Greater wing	23 (4%)
	Full lateral	158 (27.6%)
	Left	299 (52.2%)
	Pterygoid	82 (14.3%)
	Greater wing	26 (4.5%)
	Full lateral	191 (33.3%)
Lesser wing type	Right	194 (33.9%)
	Left	221 (38.6%)
Anterior type	Right	113 (19.7%)
	Left	135 (23.6%)

PSS: pneumatization of the sphenoid sinus.

**Table 3:** Relationship between PSS and PZP.

Types of PSS	PZP			*p*
	Absent (*n* %)	Present (*n* %)	Total (*n* %)	
Conchal	6 (85.7)	1 (14.3)	7 (100)	**<0.001** ^a^ (Cramer’s V = 0.285)
Presellar	14 (50)	14 (50)	28 (100)	
Sellar	109 (49.5)	111 (50.5)	220 (100)	
Postsellar	77 (24.2)	241 (75.8)	318 (100)	
			573 (100)	
Subtypes of Postsellar Pneumatization				
None	129 (50.6)	126 (49.4)	255 (100)	**<0.001** ^b^ (Cramer’s V = 0.276)
Subdorsal	43 (26.1)	122 (73.9)	165 (100)	
Dorsal	9 (19.1)	38 (80.9)	47 (100)	
Occipital	18 (23.1)	60 (76.9)	78 (100)	
Combined	7 (25.0)	21 (75.0)	28 (100)	
Lateral Pneumatization				
Absent	113 (49.1)	117 (50.9)	230 (100)	**<0.001** ^b^ (Cramer’s V = 0.225)
Present	93 (27.1)	250 (72.9)	343 (100)	
Lesser Wing Type				
Absent	128 (42.1)	176 (57.9)	304 (100)	**0.001** ^b^ (Cramer’s V = 0.136)
Present	78 (29.0)	191 (71.0)	269 (100)	
Anterior Type				
Absent	146 (36.5)	254 (63.5)	400 (100)	0.677 ^b^ (Cramer’s V = 0.017)
Present	60 (34.7)	113 (65.3)	173 (100)	

Bold *p* values indicate *p* ˂ 0.05. PSS: pneumatization of the sphenoid sinus; PZP: pneumatization of the zygomatic process. ^a^. Fisher–Freeman–Halton Exact test *p* value; ^b^. Pearson Chi-square test *p* value.

**Table 4:** Adjusted logistic regression analysis evaluating factors associated with pneumatized zygomatic process (PZP).

Independent Variable	OR (95% CI) *	*p*
PSS Type (Ref. Postsellar) *		
Conchal	0.074 (0.008–0.363)	<0.001
Presellar	0.347 (0.157–0.759)	0.008
Sellar	0.337 (0.232–0.486)	<0.001
Lateral Pneumatization	2.458 (1.719–3.515)	<0.001
Lesser wing	1.670 (1.17–2.384)	0.005
Anterior	1.056 (0.725–1.539)	0.777

* Firth’s logistic regression results. Odds ratios are adjusted for gender and age.

## Data Availability

The raw data supporting the conclusions of this article will be made available by the authors on request.

## Abbreviations

PAE	Pneumatization of the articular eminence
PGF	Pneumatization of the glenoid fossa
PZP	Pneumatization of the zygomatic process
SS	Sphenoid sinus
CT	Computed tomography
CBCT	Cone-beam computed tomography
PSS	Pneumatization of the sphenoid sinus
TMJ	Temporomandibular joint
